# Focal vs extended ablation in localized prostate cancer with irreversible electroporation; a multi-center randomized controlled trial

**DOI:** 10.1186/s12885-016-2332-z

**Published:** 2016-05-05

**Authors:** Matthijs J. V. Scheltema, Willemien van den Bos, Daniel M. de Bruin, Hessel Wijkstra, M. Pilar Laguna, Theo M. de Reijke, Jean JMCH de la Rosette

**Affiliations:** Department of Urology, AMC University Hospital, Meibergdreef 9, 1105 AZ Amsterdam, The Netherlands; Department of Biomedical Engineering and Physics, AMC University Hospital, Amsterdam, The Netherlands; Signal Processing Systems, Eindhoven University of Technology, Eindhoven, The Netherlands

**Keywords:** Irreversible electroporation, IRE, Prostate cancer, Localized, Focal therapy, Ablation, Randomized controlled trial

## Abstract

**Background:**

Current surgical and ablative treatment options for prostate cancer (PCa) may result in a high incidence of (temporary) incontinence, erectile dysfunction and/or bowel damage. These side effects are due to procedure related effects on adjacent structures including blood vessels, bowel, urethra and/or neurovascular bundle. Ablation with irreversible electroporation (IRE) has shown to be effective and safe in destroying PCa cells and also has the potential advantage of sparing surrounding tissue and vital structures, resulting in less impaired functional outcomes and maintaining men’s quality of life.

**Methods/Design:**

In this randomized controlled trial (RCT) on IRE in localized PCa, 200 patients with organ-confined, unilateral (T1c-T2b) low- to intermediate-risk PCa (Gleason sum score 6 and 7) on transperineal template-mapping biopsies (TTMB) will be included. Patients will be randomized into focal or extended ablation of cancer foci with IRE. Oncological efficacy will be determined by multiparametric Magnetic Resonance Imaging, Contrast-Enhanced Ultrasound imaging if available, TTMP and Prostate Specific Antigen (PSA) follow-up. Patients will be evaluated up to 5 years on functional outcomes and quality of life with the use of standardized questionnaires.

**Discussion:**

There is critical need of larger, standardized RCTs evaluating long-term oncological and functional outcomes before introducing IRE and other focal therapy modalities as an accepted and safe therapeutic option for PCa. This RCT will provide important short- and long-term data and elucidates the differences between focal or extended ablation of localized, unilateral low- to intermediate-risk PCa with IRE.

**Trial registration:**

Clinicaltrials.gov database registration number NCT01835977. The Dutch Central Committee on Research Involving Human Subjects registration number NL50791.018.14.

## Background

Prostate cancer (PCa) is the most common cancer in men and the second leading cancer-related cause of death. The incidence of PCa increases steadily, contributors to this increasing incidence include the ageing population, increased awareness and implementation of the Prostate Specific Antigen (PSA) test [1]. Due to the increasing use of the PSA test and improved diagnostic technology more patients with early stage, localized PCa are diagnosed nowadays.

### Present therapeutic options by guideline

Currently, there are several treatment options available for PCa, including surgery, radiotherapy, minimally invasive ablative techniques and active surveillance. Choice of treatment depends on patients’ choice and risk stratification (e.g. D’Amico Risk Classifications [2]). Active surveillance is an attractive management option for (very) low-risk PCa. Especially older men, who have the highest incidence of PCa [1], may be suitable candidates for active surveillance (AS) since these patients have a limited life-expectancy and may have co-morbidities. AS can have an impact on quality of life since patients do not receive treatment for their cancer and sometimes the window of opportunity to treat curatively can be missed. For localized disease, a radical prostatectomy or radiotherapy are the recommended curative treatments according to the guidelines [3–5]. The most common side effects associated with radical treatments are: erectile dysfunction (58–79 %), gastrointestinal toxicity and proctopathy (13–34 %), incontinence (range 3.2–14 %), overactive and/or obstructive urinary symptoms and hematuria (5 %) [6, 7]. It is the result of procedure-related damage to blood vessels, bowel, urethra and/or neurovascular bundle and may impair the quality of life in PCa patients following treatment [6].

### Focal therapy for localized prostate cancer

The side effect profile of radical treatments opened the door to focal strategies that limit damage to the important anatomical urological structures [8, 9]. A variety of ablation techniques have been used, including cryoablation, high-intensity focused US (HIFU), radiofrequency ablation (RFA), microwave coagulation, Vascular Targeted Photodynamic Therapy, Interstitial Laser Thermotherapy and Irreversible Electroporation (IRE) [10]. Focal therapy gained popularity because of the minimally invasive approach, short hospital stay, possible improved functional outcomes, lower complication rate and less impact on the quality of life [10]. Moreover, focal treatment provides a more curable treatment option compared to active surveillance and is better tolerated than radical treatments by older patients with comorbidities. A systematic review on focal treatment by *Valerio* et al. has been published, comprising a total of 2350 cases across 30 studies with a follow-up range between 0 and 11.1 year. In PCa initially treated with focal therapy, the pad-free continence ranged from 95 to 100 %, retained erectile function ranged from 54 to 100 % and PCa was absent in 50 to 96 % on regular follow up biopsies [10]. *Cheetham* and colleagues [11] published promising long-term survival rates of patients undergoing primary or salvage cryotherapy for PCa. Results indicated an 87 % overall 10-year prostate-cancer-specific survival, despite early cryotherapy technology and the majority of patients having high-risk PCa.

### Irreversible electroporation for prostate cancer

Electroporation is a technique in which high-frequent electric pulses are generated between two or more electrodes. The resultant electric current damages the cell membrane, allowing molecules to pass into the cell passively. The process of electroporation can either be temporary (reversible electroporation) or become permanent (IRE) when a certain electric threshold is reached, causing cell death due to the inability to maintain homeostasis [11–13]. Initially, the occurrence of IRE during reversible electroporation procedures was considered an unwanted side effect. However, the ability of IRE to induced selective cell death turned IRE into a tumour ablation modality, leading to the development of the commercially available Nanoknife™ (AngioDynamics, Queensbury, New York) [12]. Histopathological outcomes after IRE show a sharp demarcation between ablated and non-ablated tissue, whereas thermal ablation techniques show a transitional zone of partially damaged tissue due to insufficient temperatures for definitive ablation [14]. Therefore, IRE tissue ablation possibly enables more precise procedure planning of the target area. IRE has shown to effectively ablate tumour cells in vitro, in animal studies and in phase 1–2 human trials on IRE (cancer) ablation in several organs (liver, pancreas, kidney, lung and prostate) [15–18]. The first phase 1–2 trials on focal therapy of PCa with IRE have shown promising results, demonstrating a safe and effective focal treatment modality with low patient morbidity, improved functional outcomes and good short-term oncological control [14, 19–22]. No major complications occurred in any of these trials. All of the patients that were continent before the IRE procedure remained pad-free continent, whereas erectile function was preserved in 56 to 95 % of patients that were potent before [21, 22]. In the Phase I-II trial on IRE conducted by the Clinical Research Office of the Endourological Society (CROES) [14], IRE was performed in sixteen patients 30 days prior to their scheduled radical prostatectomy. The histopathological assessment of the prostate showed sharply demarcated necrotic and fibrotic lesions, without skip lesions [14]. This is in concordance with the other phase 1–2 trials with the exception of the study of *Valerio* et al. where 6/34 patients had possible residual tumour within the ablation zone on multiparametric Magnetic Resonance Imaging (mpMRI) [21]. In the aforementioned study, *van den Bos* et al. performed Transrectal Ultrasound (TRUS), mpMRI and Contrast-Enhanced Ultrasound (CEUS) imaging following IRE to evaluate the ability of the imaging modalities to accurately visualize IRE ablation effects, ablated area and possible residual PCa. Both mpMRI and CEUS were found to be feasible imaging modalities with concordance in volume to the ablated volume on histopathology with Pearson’s correlation of *r* = 0.88 and *r* = 0.80, respectively [23].

**Fig. 1:**
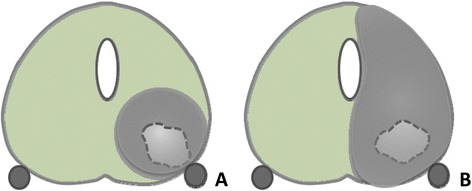
The Different Ablation Treatments. **a** Focal Ablation of a PCa lesion in Group A. **b** Extended Ablation of a PCa lesion in Group B

At present, no randomized controlled trial (RCT) (grade 2b) on IRE for treatment-naïve PCa has been conducted. Moreover, no long-term evidence is available on functional and oncological outcomes following IRE treatment in PCa. In this RCT (NCT01835977) 200 patients with histologically confirmed (on TTMB), organ-confined and unilateral low- to intermediate-risk (Gleason score 6 or 7) PCa will receive ultrasound-guided IRE. Selected candidates will be randomized to either 1) focal ablation or 2) extended hemiablation of the tumour lesions to assess the differences in side effect profile and efficacy of the treatment and to investigate if an extended ablation zone is necessary to treat undetected satellite lesions adjacent to the index lesion (Fig. 1) [24]. Patients will be evaluated on urinary continence, erectile function and quality of life with the use of standardized questionnaires [25–27]. Oncological efficacy will be determined by TTMP, preceded by mpMRI and CEUS imaging if available and PSA follow up.

## Methods/Design

### Study objectives

#### Primary

- To evaluate the differences in side effects of patients treated with focal vs extended ablation of PCa with IRE, measured by CTCAE, IIEF, IPSS and use of pads.
- To evaluate the differences in quality of life of patients treated with focal vs extended ablation of PCa with IRE, measured by EPIC, VAS pain score and length of hospital stay.

#### Secondary

- To evaluate the oncological efficacy of IRE for focal vs extended ablation of PCa, measured by TTMB 6 months after IRE and 5 year follow-up with mpMRI and serial PSA testing.
- To evaluate the utilization of mpMRI in the oncological follow up after IRE ablation of PCa and to visualize the extend of the ablation zone, performed 6 months and 1, 2, 3, 4 and 5 years following the IRE procedure.
- If available, to evaluate the utilization of CEUS in the oncological follow up following IRE ablation of PCa and to visualize the extend of the ablation zone, performed 6 months and 1, 2, 3, 4 and 5 years following the IRE procedure.

### Expected outcomes

It is expected that focal therapy with IRE in localized PCa has the potential to improve functional outcomes and the quality of life of PCa patients compared to current radical treatment. In line, it is thought that focal ablation, compared to extended ablation, could better preserve important anatomical structures and consequently have fewer side effects and a higher quality of life score. It is shown that the 2D target images produced during the preplanning procedure by the Nanoknife™ system reflect the IRE ablation zone, and could be used as a template for pathology [14]. Whole mount pathology analysis in prostatectomy specimen, obtained with radical prostatectomy 4 weeks following IRE, showed complete haemorrhage, necrosis and fibrosis in the ablated zone [14, 19]. Therefore, it is expected to find fibrosis on TTMB in the according biopsies of the planned ablated zone. Currently, no long-term oncological outcomes exist on IRE ablation of localized PCa, so it may be difficult to predict long-term outcomes. However, short-term oncological outcomes are promising and the aforementioned study showed on whole mount pathology no residual PCa in the ablated zone of all patients [14, 19–22]. It is thought that mpMRI and CEUS imaging are feasible imaging modalities for the follow-up after focal IRE ablation in localized PCa since both mpMRI and CEUS were found to accurately visualize on a short-term basis IRE ablation effects and possible residual PCa [21–23].

### Study design

**Fig. 2:**
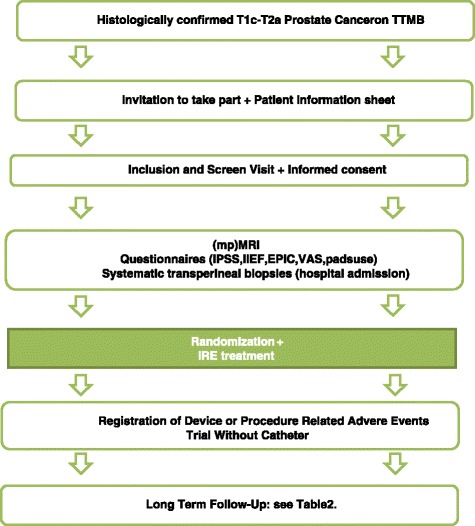
Flowchart of the inclusion and randomization process

In this prospective RCT, patients with unilateral PCa (clinical stage T1c-T2b), confirmed on transrectal ultrasound(TRUS)-guided biopsies, will be offered extensive (30-core) TTMB. If the inclusion criteria are met (see below and Table 1), candidates are randomized into two groups. One group will undergo a focal ablation of the prostate (Group A), the other group will receive an extended hemi ablation (Group B) (Fig. 1). Prior to the procedure, a baseline mpMRI, TRUS and CEUS imaging (if available) will be obtained and standardized questionnaires (International Prostate Symptom Score (IPSS), International Index of Erectile Function (IIEF), Quality of life-questionnaire (EPIC), Visual Analogue Scale (VAS)) and number of pads used will be completed by the patients (Fig. 2). After the focal or extended IRE ablation, follow-up will be performed following a strict schedule up to 5 years (Table 2) using mpMRI, TRUS and CEUS imaging (if available), blood tests (PSA, Creatinine), adverse event reporting with Common Terminology Criteria for Adverse Events (CTCAE) and the use of standardized questionnaires (IPSS, IIEF, EPIC, VAS). Six months following the IRE procedure patients will undergo TTMB to determine ablative effectiveness on pathology and to exclude any residual or ‘new’ disease. In case of any positive biopsies on follow-up TTMB, patients will be offered salvage treatment following the EAU Guidelines on Prostate Cancer [3].

**Table 1:** Inclusion and exclusion criteria

Inclusion criteria	Exclusion criteria
1. Histologically confirmed organ-confined unilateral PCa on TTMB (clinical stage T1c-T2b)	1. Bleeding disorder (prothrombin time > 14.5 s., partial thromboplastin time > 34 s.), Platelet Count <140/uL, 2. No ability to stop anticoagulant or antiplatelet therapy
2. Gleason sum score 6 or 7	3. Active (urinary tract) infection 4. History of bladder neck contracture 5. Major concurrent debilitating illness or ASA ≥4 6. Cardiac History
3. PSA <15 ng/mL or PSA >15 ng/mL counselled with caution	7. Inflammatory bowel disease 8. Major rectal surgery 9. Previous radiation to pelvis 10. Prior or concurrent malignancy
4. Life expectancy of >10 years, age ≥18 years	11. Biologic or chemotherapy for PCa 12. Hormonal therapy within last 6 months 13. Any resection or stenting of the prostate 14. Prostate calcification >5 mm

**Table 2:** Overview follow-up scheme

Visits	Day −1	Day 0; IRE	Day 1	2 weeks	4 weeks	3 months	6 months	1 year	18 months	2 years	30 months	3,4,5 years
Medical History	X											
Phys. Examination	X			X	X	X	X	X	X	X	X	X
Informed Consent	X											
PSA/Creatinine test	X			X	X	X	X	X	X	X	X	X
Questionnaires (IIEF,IPSS,EPIC,pads)	X				X	X	X	X	X	X	X	X
Pain-scores (VAS)	X		X	X	X	X	X	X	X	X	X	X
(mp)MRI and if available CEUS	X						X	X		X		X
TTMB	X						X					
IRE procedure		X										
AEs reporting		X	X	X	X	X	X	X	X	X	X	X

### Population

Two hundred patients (age ≥18 years) diagnosed with histologically confirmed, unilateral organ-confined PCa (clinical score of T1c-T2b) with positive biopsies number on transperineal template-guided prostate biopsies and no evidence of lymph node or distant metastases. Patients should have an expected life expectancy of >10 years and serum PSA <15 ng/mL or PSA >15 ng/mL counselled with caution. Patients known with a cardiac history including arrhythmias, ICD or pacemaker and patients with previous pelvic surgery or radiotherapy or PCa treatment are excluded. For all inclusion and exclusion criteria, see Table 2.

### Study procedures

#### Transperineal template-mapping biopsies

Extensive transperineal three-dimensional (3D)-template mapping biopsies are performed using ultrasound guidance to localize cancer foci by applying the same transperineal grid used for brachytherapy seed placement. This is considered to be the reference standard for patient selection in focal therapy for localized PCa [28]. TRUS biopsy, even with optimized protocols, has been shown to poorly reflect the tumour grade and extent of PCa compared with radical prostatectomy specimens [29, 30]. Needle biopsies will be analysed on diagnosis/residual/recurrent PCa and tumour grade by a specialized uro-pathologist.

#### IRE ablation

The AngioDynamics Inc. HVP-01 Electroporation System (also registered as the NanoKnife™ IRE System) is the first commercially available device based on IRE and is used for tissue ablation that is primarily cellular [13]. It is approved by the regulatory authorities in Europe (CE certificate) and meets several international recognized standards. The NanoKnife™ System and Probes have been cleared for marketing by the U.S. Food and Drug Administration (FDA) via 510(k) Premarket Notifications (K060054, K080202, K080376, and K080287). The console consists of two major components; a Low Energy Direct Current generator and needle electrodes.

**Fig. 3:**
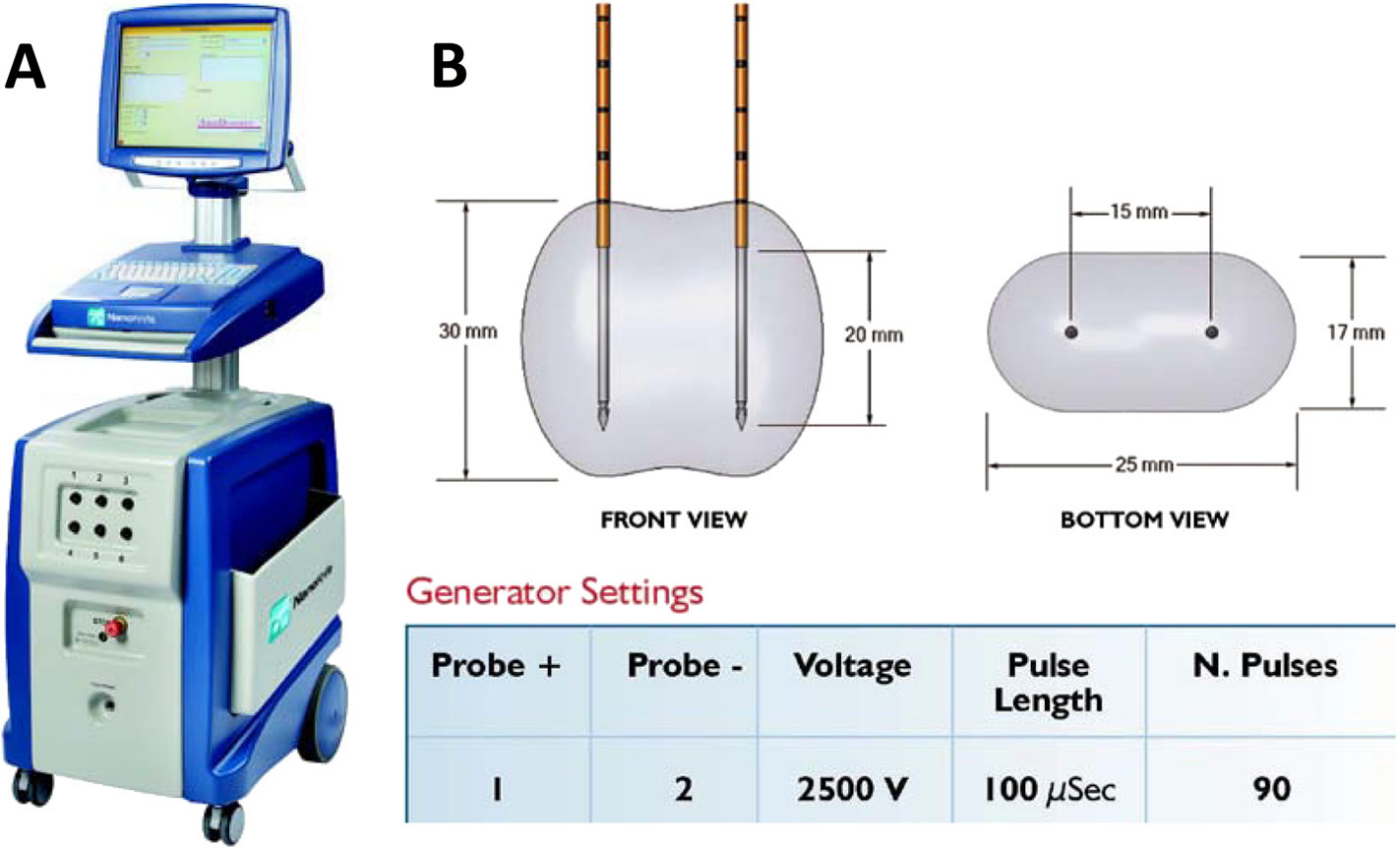
**a** The Nanoknife console. **b** Specific ablation zone: with the needles placed 1.5 cm apart, the active electrode length is set at 2 cm and the resultant ablation volume is calculated at 12.75 cm^3^

Prior to the procedure patients will receive antibiotic prophylaxis (Ciprofloxacin 500 mg). A maximum of six IRE electrodes will be placed using a brachytherapy template-grid into the pre-specified ablation zone using biplane transrectal ultrasound image guidance to visualize both sagittal and axial views. The ultrasound data on prostate dimensions, electrode position and electrode tip length are transferred into the software platform and the resultant ablation volume will be determined and displayed by the Nanoknife™ console (Fig. 3). When the needles are in place, 90 consecutive pulses will be delivered of high-voltage (1500 V/cm) with a direct current between 20 and 50 A. Pulses are delivered (synchronized with ECG) in groups of 10 at intervals of 100 μs. Between each group, a 3.5 s delay is required to recharge the capacitor. During the IRE procedure patients will receive a strong muscle relaxant (Rocuronium) to prevent severe muscle contraction. The total procedure time, including general anaesthesia, will be approximately 1 h. After the procedure patient will receive a transurethral catheter for 24 h. Patients will have an overnight hospital stay and it is anticipated that the majority of patients will be discharged the day after the procedure. Procedural and post procedure Adverse Events (AEs) and Serious AEs will be reported to the Institutional Review Board (IRB).

#### Multiparametric magnetic resonance imaging

mpMRI will be performed in supine position on a 1.5 T AVANTO® MRI scanner (Siemens Healthcare, Erlangen, Germany) using an integrated endorectal-pelvic phased-array coil (Medrad, Warrendale, USA). Prior to the mpMRI, anti-Peristaltic Drugs (Buscopan or Glucagon) will be given. T2 weighted sequences will be performed in sagittal, coronal and transverse planes from the aorta bifurcation to the pubic symphysis, including prostate and seminal vesicles. Next, T1 diffusion-weighted with fat suppression and dynamic contrast-enhanced images (0.1 mmol of gadopentetate dimeglumine per kg of body weight, Gadolinium DTPA, Gadovist) are obtained. mpMRI will be evaluated by a specialized uro-radiologist (blinded for the randomization) on prostate and ablation volume, evidence of residual or recurrent PCa according to the PIRADS (v2) criteria [31], skip lesions within the target zone, damage to adjacent structures and lymph node or haematogenous metastasis.

#### Contrast-enhanced ultrasound

CEUS imaging involves the use of microbubble contrast agent (SonoVue; Bracco, Milan, Italy) to show blood flow and tissue perfusion information. The ultrasound contrast agent consists of a solution of gas-filled shell-stabilized microbubbles with a diameter of 3–5 μm. These bubbles stay inside the blood stream and travel through all blood vessels, including the microvasculature [32] and are visualized with specialised imaging techniques (Philips iU22; Philips Healthcare, Bothell, USA). The prostate will be scanned in 4 planes, base, mid-base, mid-apex and apex. If performed, CEUS imaging will be analysed on prostate and ablation volume and evidence of residual or recurrent PCa by the performing urologist/fellow.

### Randomization, sample size and statistical analysis

#### Randomization

The patients are stratified on baseline total IIEF-score with cut-off score of 45 points (≤45 vs. >45), Gleason score (grouped in Gleason sum score 6 vs. sum score 7) and age with cut-off score of 60 years (≤60 and >60) to prevent randomly occurring differences in important prognostic factors across the two randomized groups. Patients are blinded to the randomized treatment given (focal or extended ablation) to ensure unbiased assessment. The treating physicians have to be informed about the actual treatment in order to be able to perform the ablation. mpMRI findings will be coded and centrally assessed by a uro-radiologist, blinded for the results of TTMB and the performed procedure.

#### Sample size calculation

Since the primary objective of the study is to determine the side effect profile of IRE, the sample size is powered on a common event; erectile dysfunction. *Ahmed* et al. performed three comparable studies with a concurrent focal ablation modality (HIFU), and a similar patient population and the same primary objectives [33–35]. Focal ablation with HIFU led to a decline of 18.2 % on the IIEF-15 questionnaire (57.5 % to 47.0 % after 12 months). Converting this to the shortened IIEF-5 questionnaire (which is used in this RCT), the mean IIEF score is 16.61 (SD 4.0) in Group A (focal ablation) and the mean IIEF score is 15.1 (SD 4.0) in Group B (extended ablation). A two group Wells-Satterthwaite *t*-test of equal means and unequal variances were used to calculate the sample size. The one-sided (one-tailed) test was used, because this rejects the null hypothesis for differences in a single direction. With an α level of 0.05 and power of 80 % (1-β), the sample size required was at least 90 men per group. The sample size was adjusted to allow for 35 % of men in the general population (>50 years old) having poor baseline erectile function [36], and therefore aim to recruit at least 200 men in total.

#### Statistical analysis

Data will be collected using the Data Management System of CROES. The validated questionnaires will be analysed with standard methods. Maximum and minimum values will be set according to the extremes of questionnaire item scales. Categorical outcomes will be reported as point estimates with binomial 95 % CIs to demonstrate level of precision. Wilcoxon signed rank test (two-tailed) will be used to assess differences between continuous variables that were not normally distributed (PSA and questionnaire scores), measured at baseline and at the subsequent follow-up visit. Changes over time will be reported with box-and-whisker plots. The group analyses will be hypothesis-generating and we will run statistical tests of significance.

### Quality and patient safety monitoring

Data will be centrally collected and monitored by the CROES using their Data Management System. During the study independent physicians and the IRB, assessing patient safety and treatment efficacy, will monitor data. Monitoring criteria are objectively described in the protocol approved by the IRB, since IRE ablation of soft tissue is a novel prostate cancer treatment and therefore holds potential risks and side effects that are unknown at this time. A clinical risk analysis associated with the NanoKnife™ device and the procedure is presented in Table 3. In accordance to section 10, subsection 1, of the Wet Medisch-Wetenschappelijk Onderzoek met Mensen (Medical Research Involving Human Subjects Act), the investigator will inform the subjects and the reviewing accredited IRB if anything occurs of which the disadvantages of participation may be significantly greater than was foreseen in the research proposal. The study will be suspended pending further review by the accredited IRB, except insofar as suspension would jeopardise the subjects’ health. The local investigator will inform all participating subjects. Interdepartmental monitoring (IDM) will take place according to the IDM model in the AMC.

**Table 3:** A clinical risk analysis associated with the IRE device and procedure

Potential risks of IRE ablation and procedure	Side effect/Adverse event
General Anaesthesia	Aspiration, urinary retention, extended muscle blockage, anaesthetic drug toxicity, pain, coma, death
Electric current of IRE	Cardiac arrhythmias, severe muscle contraction, electrical shock, death
Multiple Prostate Biopsies	Bleeding, infection, pain, urinary retention, pain
IRE needle placement and ablation	Damage to urethra/bowel/bladder/nerve with consequent side effects^a^, bleeding, infection, pain
Insufficient IRE treatment	Residual or recurrent tumour
Insufficient Muscle Blockade	Muscle strains or damage, electrodes displaced, trauma

^a^haematuria, hematoma, infection, abscess formation, fistula, sepsis, death, urinary retention, urinary or faecal incontinence, urethra stricture, erectile dysfunction, necrosis of affected tissue

### Ethical consideration

This RCT will be conducted in accordance to the standards of Good Clinical Practice, with the ethical principles that have their origins in the Declaration of Helsinki (Fortealeza, Brazil, October 2013) and is approved by the IRB of the Academic Medical Center, Amsterdam (2014_303). The protocol is registered with The Dutch Central Committee on Research Involving Human Subjects (NL50791.018.14) and on the clinicaltrials.gov database (NCT01835977). Potential patients will be informed about the study by one of the principal investigators or its representatives, provided with the patients information form. When patients agree to participate, written informed consent is acquired from all participants.

### Availability of data and materials

The study initiator, international coordinating researcher and biostatistician will have access to all data in the CROES system, participating centres will only be able to access and register their own data. All data is available for audit and all data will be published in an international peer-reviewed medical journal. After the finalization of the study, the dataset supporting the conclusions of this study will not be shared since permission was not obtained from all participating centres.

## Discussion

The first phase I-II trials on focal therapy of PCa with IRE have shown promising results for IRE as a safe and effective focal treatment modality with low patient morbidity, improved functional outcome and good short-term oncological control [14, 21, 22]. However, there is a need of larger, uniform randomized controlled trials evaluating long-term oncological outcomes to make IRE an accepted and safe therapeutic option in PCa. This study will be the first RCT (Grade 2b) with IRE for treatment-naïve, unilateral low- to intermediate risk PCa, evaluating patients’ functional outcome, quality of life and oncological control following focal (group A) or extended (group B) ablation. Two hundred patients will be included and followed up to 5 years with the use of standardized questionnaires, mpMRI, CEUS imaging if available, TTMB and serial PSA testing.

In this trial TTMB is used to select patient for focal therapy in localized PCa, as recommended after an international consensus meeting [28]. However, special computer models of 3D whole mount prostatectomy specimens were compared in a small series (*n* = 25) to their corresponding TTMB cores. Eighteen out of sixty four lesions were missed (only 1 was clinically significant) by TTMB and sum Gleason score was upgraded in 12 % (*n* = 3) [30]. A concurrent study by *Hu* et al. showed the superiority of TTMB above TRUS-guided biopsies in diagnosing and staging PCa but TTMB regimen still missed 5 % of ≥0.2 mL and ≥0.5 mL lesions [29]. This highlights the confounding factor that TTMB has on accurate patient selection, treatment zone determination and oncological follow-up. In line, PCa foci may also be missed on mpMRI. mpMRI data (on a 1.5 T device) compared to whole mount prostatectomy specimen showed sensitivity/specificity and positive/negative predictive values for detection of PCa by mpMRI of 77/91 and 86 %/85 % for foci of >0.2 mL and 90/88 and 77 %/95 % for foci of >0.5 mL, respectively [37, 38]. However, in a recent study (*n* = 50) the combination of mpMRI with TTMB, used in a RCT, was found to have a high negative predictive value (91 %) for low-grade, small volume (>3 mm) PCa, indicating a reliable assessment to prevent undertreating significant PCa [39]. Since pathological assessment following IRE showed fibrosis without glandular prostatic ducts and necrotic tissue and no skip lesions within the ablated zone, we believe IRE effectively ablates all PCa foci within the target zone. Tumour ablation experiments in animals and humans have shown the potential to preserve connective tissue structures within the target zone and limit damage to associated blood vessels, neural tissue or other vital structures [16–18]. However, this may not apply to current thorough clinical treatment protocols [14, 40].

Therefore, in our opinion this RCT will provide important data on the differences between focal or extended ablation of localized, unilateral low- to intermediate-risk PCa using IRE, providing essential long-term data on functional outcome and oncological control.

## Abbreviations

(mp)MRI: (multiparametric) Magnetic Resonance Imaging
A: ampere
AE: adverse event
AS: active surveillance
CEUS: Contrast-Enhanced Ultrasound
CROES: Clinical Research Office of the Endourological Society
CTCAE: Common Terminology Criteria for Adverse Events
EPIC: Quality of life-questionnaire
HIFU: high-intensity focused ultrasound
IDM: interdepartmental monitoring
IIEF: International Index of Erectile Function
IPSS: International Prostate Symptom Score
IRB: Institutional Review Board
IRE: Irreversible Electroporation
PCa: Prostate Cancer
PSA: Prostate Specific Antigen
RCT: randomized controlled trial
RFA: radiofrequency ablation
SAE: serious adverse event
SD: standard deviation
TRUS: Transrectal Ultrasound
TTMB: transperineal template-mapping biopsies
V: Volt
VAS: Visual Analogue Scale
